# Phosphide of Zinc

**Published:** 1880-01

**Authors:** 


### PHOSPHIDE OF ZINC.

Phosphide of zinc has proven a most efficient agent in the successful treatment of a certain class of affections. In very many instances it has been far more curative than phosphorus. Considered in the light of a curative agent, the phosphide of zinc stands alone, not only for the certainty, but for the rapidity of its action as a nervous tonic and stimulant. Its value, in these respects, has of late been fairly tested in the last and exhaustive stages of typhoid and other fevers, where the nervous energies have been so far prostrated as to render convalescence, if not doubtful, at least tedious and protracted. The great therapeutic value of the phosphide is evinced in the most distinct manner when used in the treatment of neuralgia. While phosphorus is seldom curative in doses of less than one-twentieth or one-tenth of a grain, phosphide of zinc yields as reliable and more speedy results in doses of one-tenth to one-eighth of a grain. Few stomachs can tolerate more than one-thirtieth of a grain of phosphorus before manifesting symptoms of irritation, which, in connection with the "matchy" taste soon evolved in eructations, often engender a disgust to its further continuance. On the other hand, experience with the phosphide of zinc has proven that it enters the circulation far more readily than the element, and in doses of from one-eighth to one-twelfth of a grain produces its curative influence far more rapidly, and is equally as permanent in therapeutic power.

It has been found extremely serviceable in neuralgia, in angina, in loss of memory and impotence, in loss of sleep from combined mental anxiety, and generally in those nervous affections that owe their origin to exhaustion and depression of the nerve force. Dr. Hammond's formula is one-sixteenth grain phosphide of zinc with one-fourth grain of ext. tinct. vomica, made into a pill.

